# Leg Regrowth in *Blaberus discoidalis* (Discoid Cockroach) following Limb Autotomy versus Limb Severance and Relevance to Neurophysiology Experiments

**DOI:** 10.1371/journal.pone.0146778

**Published:** 2016-01-29

**Authors:** Timothy C. Marzullo

**Affiliations:** Research and Development, Backyard Brains, Inc., Ann Arbor, Michigan, United States of America; University of Szeged, HUNGARY

## Abstract

**Background:**

Many insects can regenerate limbs, but less is known about the regrowth process with regard to limb injury type. As part of our neurophysiology education experiments involving the removal of a cockroach leg, 1) the ability of *Blaberus discoidalis* cockroaches to regenerate a metathoracic leg was examined following autotomy at the femur/trochanter joint versus severance via a transverse coxa-cut, and 2) the neurophysiology of the detached legs with regard to leg removal type was studied by measuring spike firing rate and microstimulation movement thresholds.

**Leg Regrowth Results:**

First appearance of leg regrowth was after 5 weeks in the autotomy group and 12 weeks in the coxa-cut group. Moreover, regenerated legs in the autotomy group were 72% of full size on first appearance, significantly larger (*p*<0.05) than coxa-cut legs (29% of full size at first appearance). Regenerated legs in both groups grew in size with each subsequent molt; the autotomy-removed legs grew to full size within 18 weeks, whereas coxa-cut legs took longer than 28 weeks to regrow. Removal of the metathoracic leg in both conditions did not have an effect on mortality compared to matched controls with unmolested legs.

**Neurophysiology Results:**

Autotomy-removed legs had lower spontaneous firing rates, similar marked increased firing rates upon tactile manipulation of tibial barbs, and a 10% higher electrical microstimulation threshold for movement.

**Summary:**

It is recommended that neurophysiology experiments on cockroach legs remove the limb at autotomy joints instead of coxa cuts, as the leg regenerates significantly faster when autotomized and does not detract from the neurophysiology educational content.

## Introduction

Our research group studies insect neurophysiology for educational applications, where typical experiments involve cutting the leg off of a cockroach through a transverse coxa-cut, inserting electrodes into the coxa and tibia/tarsus joint, and monitoring the neural activity over a number of hours in the still-living detached leg [1–3]. As cockroaches can survive the leg removal and continue to eat, locomote, and breed with 1–2 legs missing, a question we often receive from the scientific community and the public during demonstrations is whether the leg can regenerate in our experiments.

Invertebrates are famous for their regenerative abilities, notable examples including the sea star and the planarian. Indeed, this is no different for insects, with many using leg autotomy as a defense mechanism against predators [4]. The first reports of invertebrate limb regeneration in general were by René-Antoine Réaumur in 1734 [5] on crabs and certain insects, and the first report on cockroach limb regeneration in particular was by Harold Brindley in 1897 [6]. The cockroach limb regeneration was discovered by accident when Brindley and his colleagues noted that 1–2 random legs of any given cockroach (in *Peripleneta americana* and *Blattella germanica*) have only 4 tarsal joints instead of the normal 5. Investigation revealed that a leg with only 4 tarsal segments was in fact a regenerated leg.

Since then, it has been borne out by scientists in the 20^th^ century that epidermal cells respond to the limb injury and develop a new leg *de novo* from a blastema, and this leg regeneration can delay a subsequent molt by 1–3 days [7–8]. The cockroach leg has built-in autotomy planes at both the femur/trochanter joint and the tibia/tarsal joint, which allow the limb to separate without substantial injury to the animal. Regenerating legs appear when animals molt, and grow in size with each subsequent molt.

Though cockroach leg regrowth has been studied by multiple groups over the past 118 years, almost all the work has focused on removal at the two autotomy joints in newly hatched cockroaches [6–13] that are too small (<5 mm in length) for our neurophysiology experiments. Moreover, the effect of leg removal method on the subsequent neurophysiology of the detached leg has to date been unstudied.

**Fig 1:**
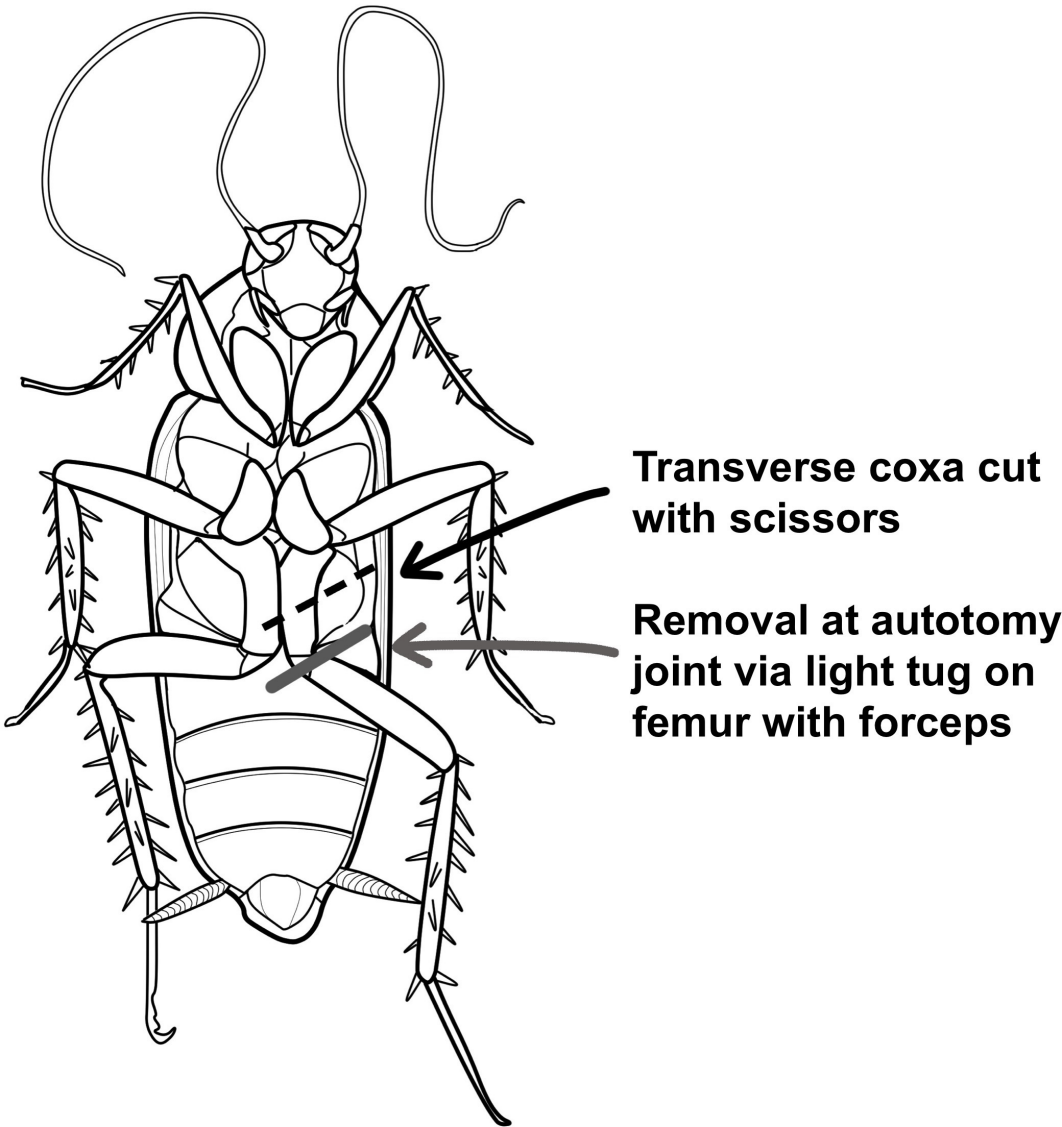
The two different types of leg removal utilized.

To determine the time course of leg regeneration as regards to leg removal type in juvenile cockroaches, survival and leg regrowth was studied in *Blaberus discoidalis* (3–6 months of age, 20–30 mm in length) in which the leg was removed either through a) autotomy at the femur/trochanter joint (as the regeneration literature has studied [6–13]), or b) a transverse cut through the mid-coxa (as the neurophysiology community uses [1, 3]) (see **Fig 1**). Furthermore, the neural spiking firing rate activity and microstimulation thresholds in autotomy-removed versus coxa-cut legs was studied to examine neurophysiology changes dependent on leg removal type.

The thorax of the cockroach contains three ganglia, each one receiving sensory input and sending motor output to its corresponding pair of legs (prothoracic, mesothoracic, and metathoracic). Each side of each ganglion has 7 nerves that are numbered in rostral to caudal order [14–15]. Four of these nerves (numbers 3, 4, 5, and 6) subsequently innervate the leg. Through detailed anatomical studies [16–18], it has been found that nerves 4 and 6 are purely motor, while nerves 3 and 5 contain a mix of both sensory and motor fibers. Nerves 3, 4, and 6 innervate the coxa and trochanter structures, whereas nerve 5 is the only nerve whose trunk and ramifications innervate the femur, tibia, and tarsus of the leg.

The motor axons control the various leg musculature, and the sensory axons deliver information from the leg’s propriosensors: 1) the chordotonal organs and hair plates that detect the relative position of the joints and 2) the campaniform sensilla which sense deformation of the leg cuticle as well as the prominent tibial barbs [14, 19].

## Materials & Methods

*Blaberus discoidalis*, popularly called “discoids” or “false death’s head cockroaches,” were used for this work. Though discoids have not been previously studied for their leg regeneration abilities, they are commonly used by scientists studying biomechanics, motor control, and neurophysiology [20–22]. They are among the larger species of South American cockroaches (up ~75 mm in length as adults) and live under the bark of rotting trees in the Amazonian rainforest.

216 juvenile, mixed-sex, 24 ± 5 mm (s.d) in length, 0.9 ± 0.5 g (s.d) in weight individuals were randomly split into four groups of 54 cockroaches each. The four groups were 1) control, 2) autotomy, 3) coxa-cut sterilized and 4) coxa-cut unsterilized.

The control group cockroaches were placed in a tub of ice water to anesthetize them. One by one, each cockroach was removed from the ice water, its body length (from the tip of head to the end of abdomen) was measured with digital calipers, and a mark of red paint (Revlon finger nail polish) was applied to the dorsal exoskeleton as an indicator for subsequent molting. The cockroach was then placed in a communal terrarium with the rest of the control cockroaches.

The “autotomy” group was similarly anesthetized in ice water, painted, and measured. To remove the left metathoracic leg, a pair of forceps was used to grasp the mid-point of the femur of the leg, and the femur was then tugged to cause the leg to separate at the trochanter/femur autotomy joint (see **Fig 1)**. In between animals, the forceps were sterilized by dipping in ethanol and then passing through a butane lighter flame. Care was taken to remove the leg in the same manner in every individual. The autotomy cockroaches were housed together in their own communal terrarium.

There were two “coxa-cut” groups in which the left metathoracic leg was cut transversely though the midpoint of the coxa with surgical scissors. In one group, the scissors were sterilized between cuts (in the same manner as the forceps above in the autotomy group), and in another, the scissors were not sterilized. This was done to measure the effect of insect survival due to sterilization techniques, based on observations from previous pilot studies not reported here. The coxa-unsterilized group and the coxa-sterilized groups were housed separately in their own sub-group communal terrariums.

Animals were given 3 days to recover from the leg removal procedure, and then measurement sessions commenced. In each group, cockroaches were removed one by one from their home cage and examined for whether the red paint mark was present on the dorsal side. If the paint mark was absent, the cockroach was considered “molted” and placed in ice water for anesthesia and subsequent measurement (pilot studies revealed that the paint mark did not come off easily with cockroach grooming or other behaviors). If a cockroach still had its mark of red paint present, it was placed in a temporary “counting” terrarium. If a cockroach was dead, it was counted as “perished” and disposed of. After all cockroaches were accounted for, the unmolted cockroaches were then placed back in the home terrarium. Measurements of the anesthetized (recently molted) roaches were then done.

**Fig 2:**
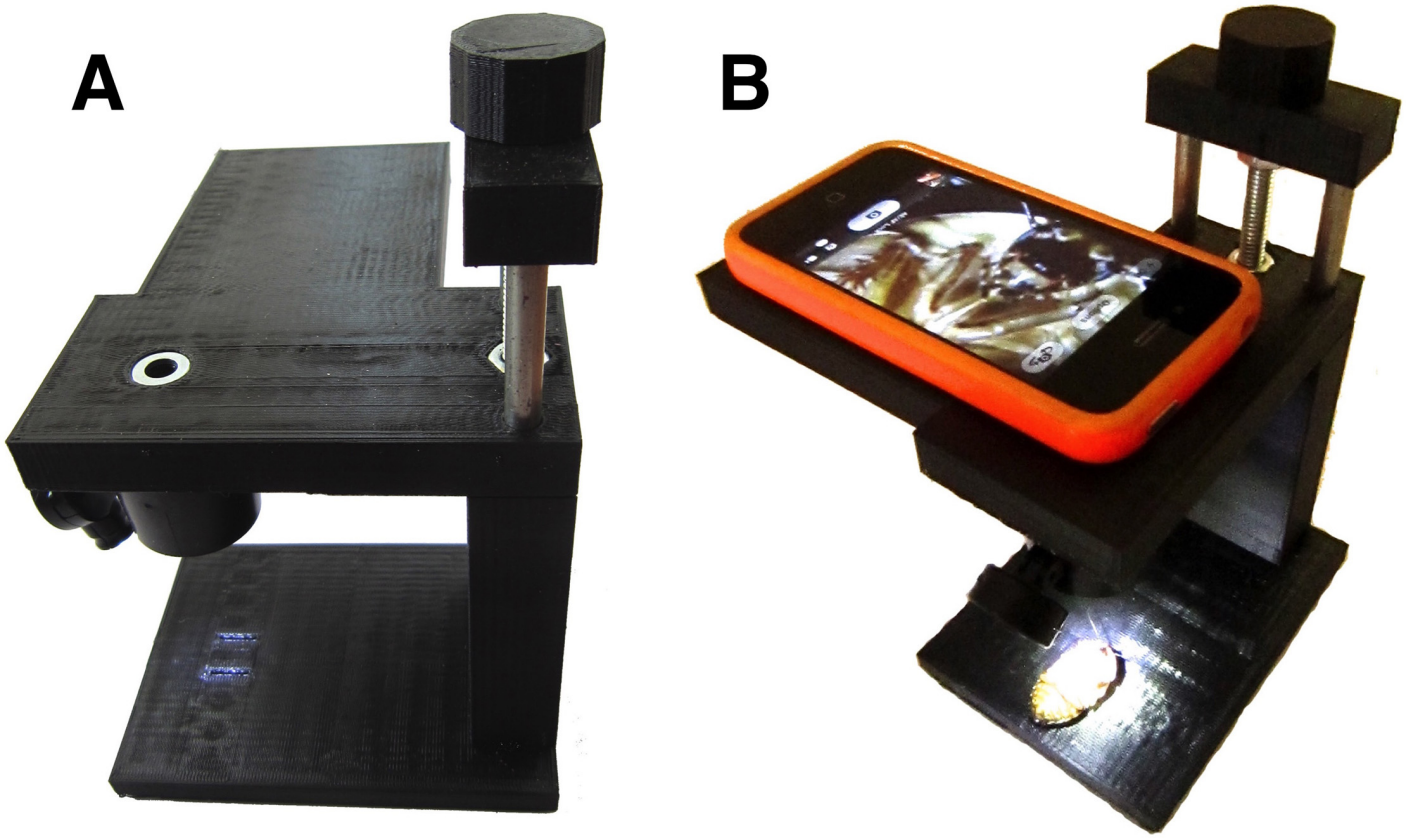
Roachscope. **A**. Side view of “RoachScope” custom 3D printed microscope with integrated 10x loupe lens. **B.** Placement of smartphone on microscope to take stable photographs.

One by one, each anesthetized cockroach was removed from the ice-water, dried, the body length was measured with digital calipers, and a photo of the ventral side of the autotomy and coxa-cut groups was taken with a custom 3D-printed 10x smartphone microscope “RoachScope” (see **Fig 2**) to record the presence or absence of a regenerated leg. When a cockroach regenerating leg first manifests, it appears “fully formed,” though in miniature, after a certain number of molts [6–13]. Thus, the two measures taken were:

1. Presence or Absence of a Leg
2. Size of Leg, if Present

This permitted the observation of healing time course, leg size, and the different growth rates during the measurement sessions in the various groups.

After photography, a fresh mark of red paint was applied to the dorsal side of the cockroach, and the cockroach was then placed back in its home terrarium. This measurement procedure was done once every week throughout the duration of the study (28 weeks in total).

To normalize within-colony effects, all terrariums were kept together in the same environment where the temperature ranged from 21–27° C (~70–80° F) during the day to 16–21°C (60–70° F) at night. The terrariums (20 x 10 x 5 cm) had approximately 5 cm of earth inside and were filled with various egg crates and cardboard paper rolls. The cockroaches were sustained with iceberg lettuce, slices of fresh potatoes and carrots, and dry cat food. Once a week, cages were cleaned and fresh food added.

To prevent breeding during the experiment, when a final molt was observed (wings present) during a measurement, such mature adults were then removed from their respective colonies and relocated to an unrelated colony to be used for other experiments and breeding of stock.

### Data Analysis

#### Survival rates of cockroaches over time

To examine whether different types of leg removal affected vitality of the cockroaches, it was noted how many cockroaches were living versus expired during each measurement session throughout the duration of the experiment.

#### Regenerating leg observation and respective size over time during molts

**Fig 3:**
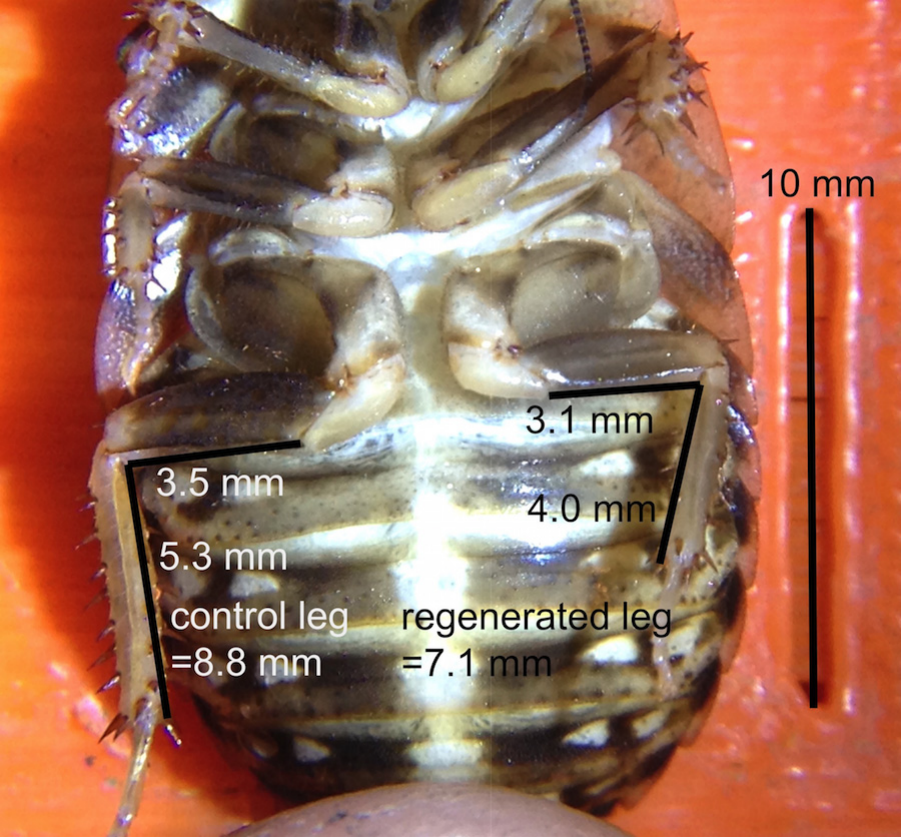
Cockroach Leg Measurement. Example of measurement of cockroach leg photographs using 10 mm reference guides on microscope base and the NIH ImageJ analysis program.

Each photo taken of the cockroaches had a built-in calibration mark of 1 mm x 10 mm (see **Fig 3**), which permitted observation and quantitative measurements of regenerated legs using the freely available NIH ImageJ analysis program. Size of the regenerating leg (left metathoracic leg) was compared to the size of contralateral uncut leg (the right metathoracic leg). The specific measurements were 1) *femur length*–measured from the medial side of the femur going from the trochanter/femur joint to the femur/tibia joint, and 2) *tibia length*–measured from the medial side of the tibia going from the femur/tibia joint to the tibia/tarsus joint (see **Fig 3**for clarity of these measurements). These two measurements of femur length and tibia length were combined to get a measure of the limb size.

### Neurophysiology Experiments

#### Neural Recording

To test the effect of the presence or lack of a coxa on the neural spiking activity of the cockroach leg, the left, metathoracic leg from 8 cockroaches was removed via a transverse coxa cut. One stainless steel “map pin” electrode was inserted into the coxa, the other map-pin electrode in the tibia/tarsus joint, and the electrodes were plugged into a “SpikerBox” Neural Amplifier [1]. 10 seconds of spontaneous activity were recorded, followed by 10 seconds of lightly tapping the prominente barbs on the tibia of the cockroach leg with a plastic probe. After this measurement, the electrode that was in the coxa was then inserted into the center of the femur, and the same recording procedure repeated (10 seconds of spontaneous activity followed by 10 seconds of light touch). Finally, the coxa was then removed with forceps, the electrodes left in place, and the recording procedure repeated once more. The spike firing rate of the spontaneous and barb deflection neural data in the three experimental conditions was then analyzed by extracting neural action potential (spike) times using a threshold window discriminator in the Backyard Brains “SpikeRecorder” software, exporting the spike times to Matlab (Mathworks, Inc., Natick, MA), and then measuring spike firing rates during the 10 seconds of spontaneous activity versus the ten seconds when the tibial barbs were deflected with a plastic probe. Averages were taken across all 8 legs in the various electrode configurations, and the Student’s t-test was used to statistically compare the results.

#### Electrical Microstimulation

In our standard microstimulation experiments [1], electrical signals of various frequencies are sent to the cockroach leg, and the movement thresholds (amplitude of the electrical signal which causes visible cockroach tibia deflection) can be observed. Previous work on insect legs by our research group has found the frequencies with the lowest microstimulation thresholds to be 50 Hz and 100 Hz [23], and a similar procedure was utilized here.

The left, metathoracic leg of 8 additional cockroaches was removed in the same manner as stated above. One map-pin electrode was placed in the coxa, and the other map-pin electrode was then inserted in the femur (the electrode cannot be placed in the tibia/tarsus joint, as was done in the neural recording experiments, as the cockroach tibia needs to be able to freely move in response to the electrical microstimulation). During the microstimulation experiments, the threshold of movement to 50 Hz and 100 Hz sine wave electrical signals was tested using the “Tone Generator” App on an iPhone 5s. An iPhone has 16 discrete volume settings (going up to maximum ~1.3 V), and the lowest volume setting that caused visible deflection of the cockroach leg at 50 Hz and 100 Hz was recorded. Following these measurements, the electrode in the coxa was moved into the femur (both electrodes now in the femur), and the thresholds for movement to 50 Hz and 100 Hz were observed similarly. Finally, the coxa was removed with forceps, and the thresholds measured a third time. The thresholds in all 8 legs were averaged for the three experimental conditions, and the Student’s t-test was performed to examine statistical difference between the groups.

## Results & Discussion

### Survival Rates

Of the 216 cockroaches at the beginning of the study, 152 (70%) were still living at the close of the study (28 weeks). Cockroach death was not related to injury type; chi-square tests did not reveal any significant differences in vitality (percentage living) between the four groups (control, autotomy, coxa-cut unsterilized, and coxa-cut sterilized) during the duration of the study (p = 0.82, df = 3, N = 216). Due to this lack of observed difference, all major subsequent analysis in this manuscript shall treat the 52 unsterilized and 52 sterilized coxa-cut cockroaches as one group of 104 individuals.

Though removal of a single metathoracic leg does not affect vitality compared to control cockroaches, this experiment was done in a controlled environment where all cockroaches had *ad libitum* access to food and no insectivorous predators were present. In natural environments where resources are scarce, the lack of a leg may reduce the competitive fitness of the cockroach (affecting speed and navigation), as it is known cockroaches use the tarsal structures on their metathoracic legs to spin around ledges during escape behavior [24].

### First Leg Appearance and Molting Frequency

**Fig 4:**
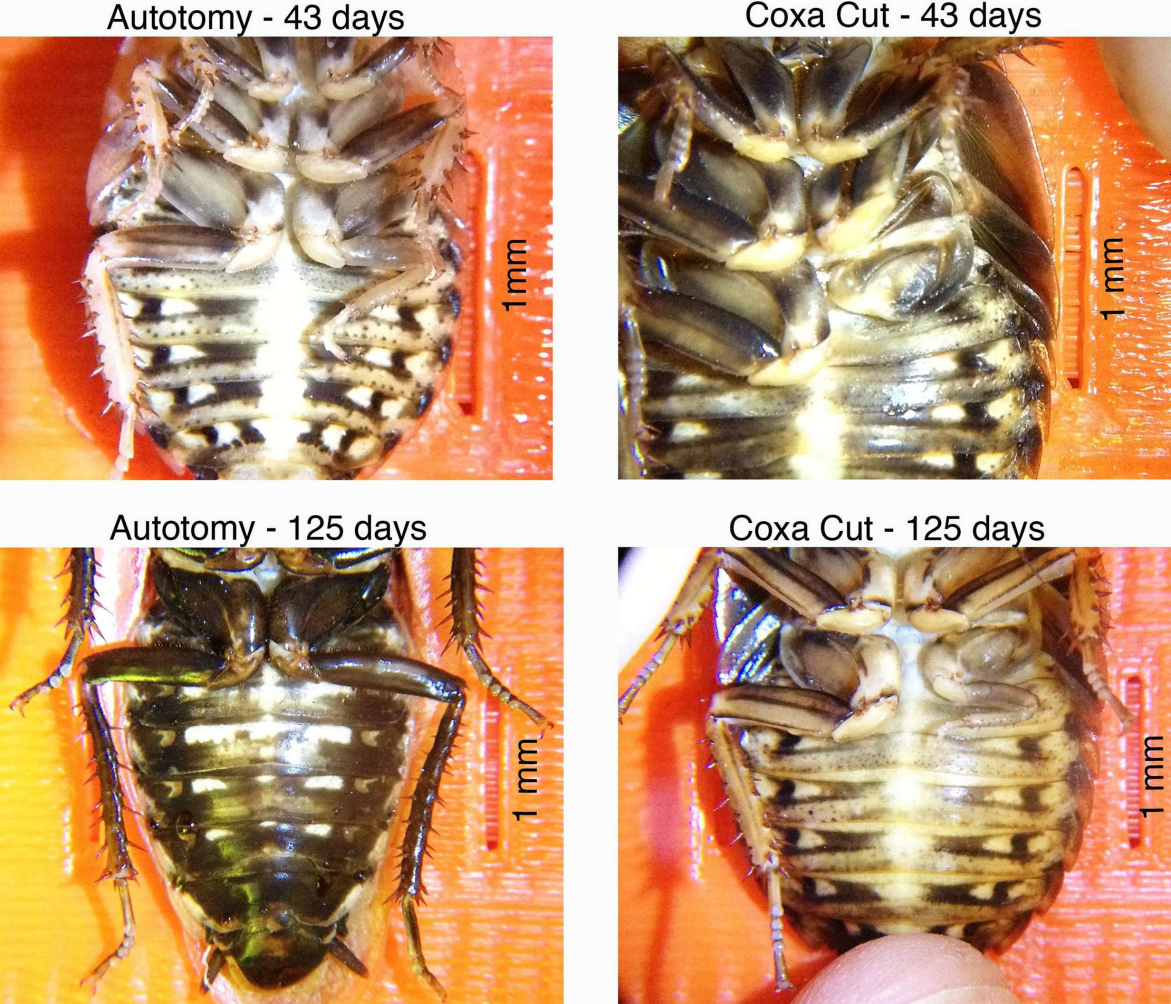
Observation of cockroach legs at various time points. Note that at 43 days a well-formed regenerated leg exists in the autotomy cockroach, but the coxa-cut cockroach only reveals a healed coxa and lacks a regenerated leg. At 125 days, the autotomy-removed leg has fully regrown, but in the coxa-cut leg, the regenerated leg is still quite small. Images over time are not necessarily of the same cockroaches.

**Fig 5:**
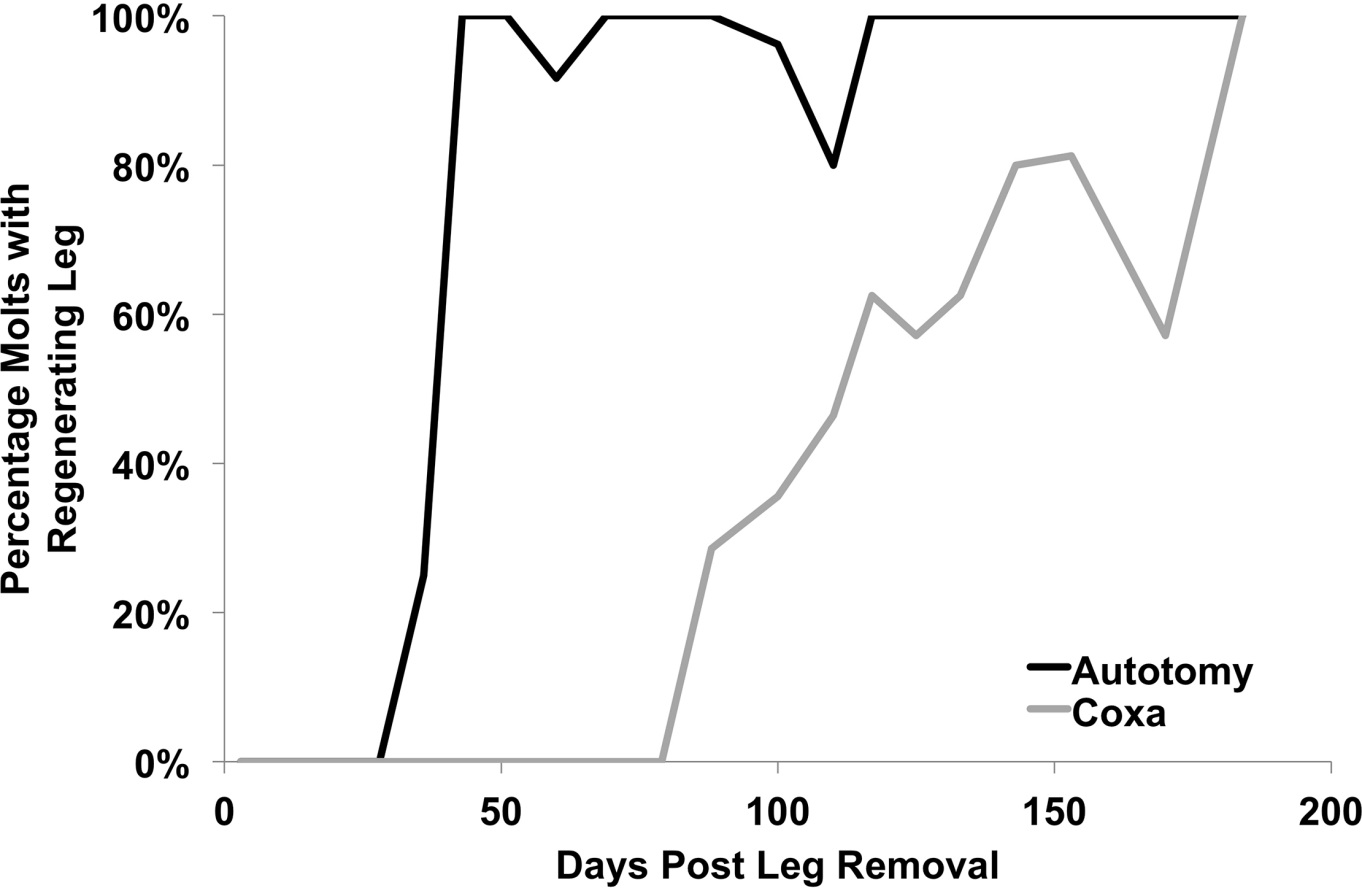
Percentage of new molts with a regenerated leg. Y–axis refers to the percentage of molts observed at the indicated time point that revealed a regenerated leg. After day 43 post-leg removal, in the autotomy group, almost all molts contained a regenerated leg. Similar observations were delayed in the coxa-cut group. The dip at day 110 in the autotomy group (12 of 15 observations showing a regenerated leg) and at day 170 in the coxa-cut group (4 of 7 observations showing a regenerated leg) is likely due to molt failure, with the already regenerating leg self-autotomizing during the molt.

See **Fig 4**for photographic examples of regenerated legs and **Fig 5**for the aggregate time course of regenerated leg appearance in the populations. Over the course of the study, 152 molts occurred in the autotomy group (from N = 54 cockroaches), of which 120 molts showed a regenerating leg (78%). In the combined coxa-cut group, 273 molts total occurred (from N = 108 cockroaches), of which 80 demonstrated new legs (29%). This significant difference in percentage (chi-square test, p <0.0001, df = 1, N = 425) is due to the delayed regeneration of the coxa-cut legs. First observation of a new leg in the autotomy group was at 5 weeks, and, considering only the molts that occurred after the first observation of a regenerated leg, almost all subsequent observed molts showed a new leg (94%, of 128 molts). The appearance of new legs in the autotomy group thus followed a “step function” pattern, with new legs appearing at roughly the same time in the population following a healing process lasting approximately 5 weeks. In the coxa-cut group, the appearance of new legs was more gradual, with the percentage of observed molts showing a regenerated leg progressing in a linear fashion after new legs began appearing at 12 weeks (see **Fig 5**). For example, of the 148 molts that occurred after the first observed leg regrowth in the coxa-cut population, only 54% showed a regenerated new leg.

### Size of regrowing legs

**Fig 6:**
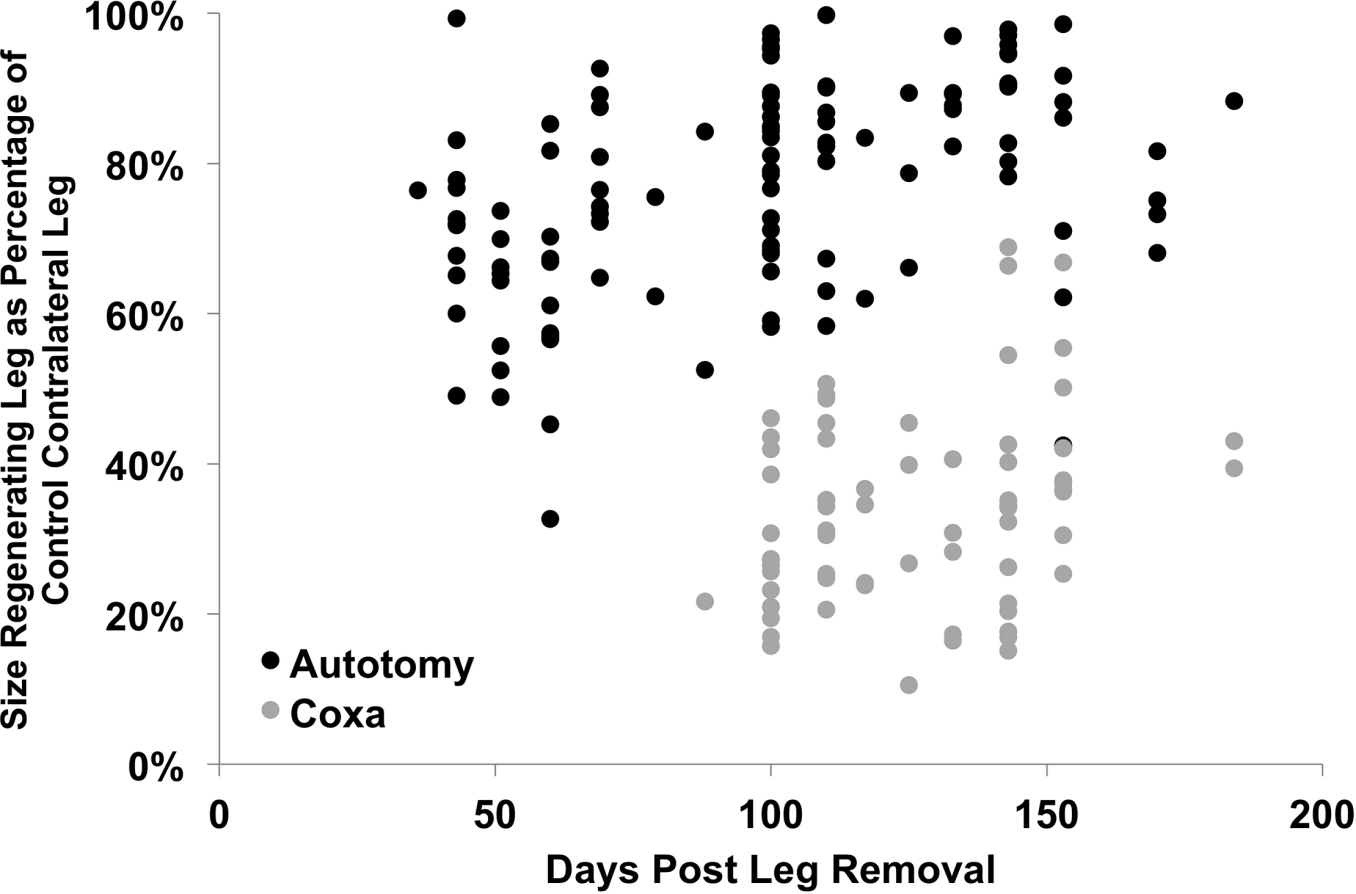
Size of regenerated leg in comparison to contralateral uncut control leg. In molts where a regenerated leg was observed, the size of the femur and tibia were measured on both the regenerated leg and the control leg. Femur and tibia length were then added together, and the size of the regenerated leg as a percentage of the length of the contralateral control leg was calculated. Autotomy-removed legs regrow earlier and are larger than coxa-cut legs.

See **Fig 6**for an analysis of the size of the regenerating leg in comparison to the contralateral uncut leg. Autotomy legs were always larger than the coxa-cut legs at any given time point. For example, at 43 days, when regenerating legs began appearing regularly (>33% of observations showing new leg, number of new leg observations >2) in the autotomy group, the mean autotomy leg size was 72% of the contralateral control leg (12 observations). When legs began appearing regularly in the coxa-cut group (>33% of observations showing new leg, number of new leg observations >2), at 100 days the new leg was only 29% of the size of the contralateral control leg (14 observations), significantly smaller than the autotomy-regenerated legs (student’s t-test: p <0.0001, df = 24, N = 26) and also later in appearance. In both groups, subsequent molts revealed increasingly larger legs with time. In the autotomy group, full leg regrowth (considered to be at 80–100% size of the contralateral control leg) took place within 100–150 days (**Fig 6**), whereas in the coxa-cut group, full leg regrowth required more than 200 days.

The likely reason for the more efficient recovery in the autotomy group is that an autotomy-removed leg more closely resembles an injury the cockroach would actually experience in its natural environment, as random cockroaches in the field can often have 1–2 legs missing at autotomy points due to predation or other noxious events [6]. It is rather challenging to imagine a situation where a cockroach would suffer an injury or attack resulting in a transverse coxa-cut that it would subsequently escape and recover from. Thus, there was likely less evolutionary pressure to develop mechanisms to recover rapidly from coxa-cut injuries than autotomy point injuries. Examining regeneration abilities between invertebrates that have limbs which extend laterally (such as various crustaceans and arachnids), and are more vulnerable to attack, and invertebrates that have limbs which extend ventrally (such as termites and cockroaches) may reveal differences in speed of regeneration from non-autotomy breakage points.

### Neurophysiology Experiments

**Fig 7:**
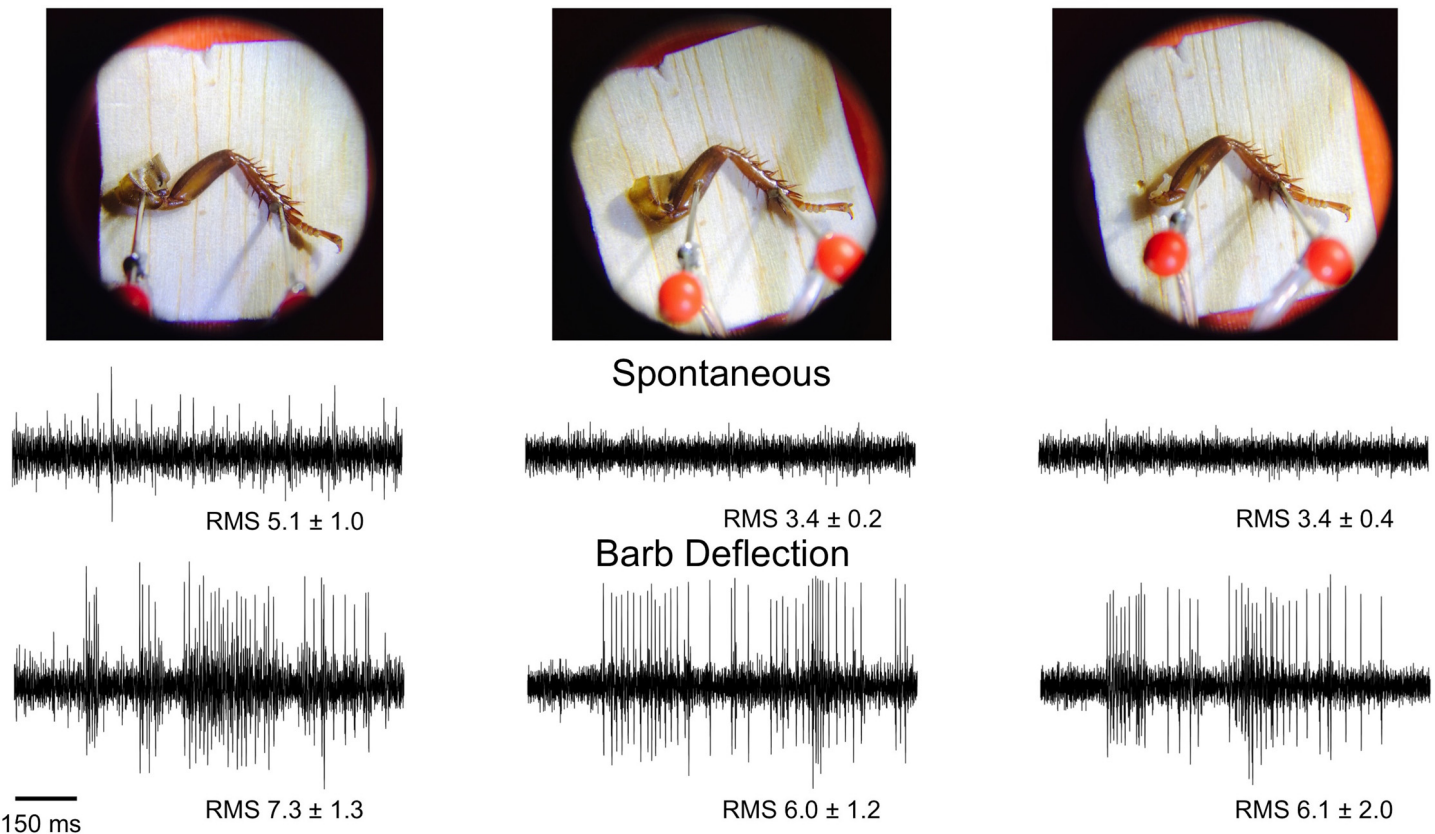
Neural Recordings in Different Electrode Configurations. Electrodes were first placed in the coxa and tibia tarsus joint; spontaneous rate was recorded for 10 seconds, followed by 10 seconds of lightly touching the tibial barbs with a plastic probe. The coxa electrode was then moved to the femur, and a similar recording of spontaneous activity and tactile activity done. Finally, the coxa was removed with forceps, and the measurements of neural signals repeated. The average root mean square, a measure of signal amplitude, for all 8 legs is shown underneath the traces and is reported in arbitrary A/D (analog to digital) conversion units.

**Fig 8:**
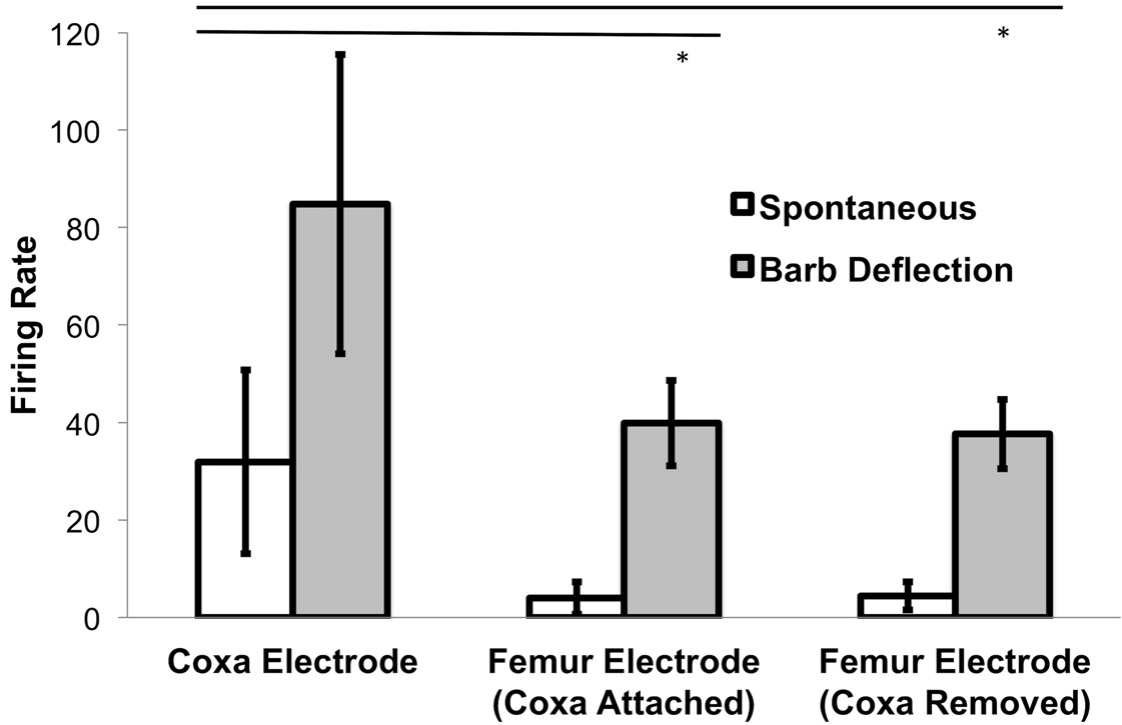
Firing Rate Analysis in Different Electrode Configurations. Firing rate was measured during 10 seconds of spontaneous activity followed by 10 seconds of evoked spiking (by manually deflecting the tibial barbs) during all three conditions. Firing rate average and standard deviation from all 8 legs measured is shown (same data set as shown as **Fig 7**). Spontaneous Firing Rate and Barb Deflection Firing Rate was significantly higher when one electrode was in the coxa versus when one electrode was in the femur. No differences were observed between the coxa attached and coxa removed femur electrode conditions. * = p<0.05, Student’s t-test.

See **Fig 7**for sample electrophysiology traces, electrode configurations, and average root mean square (RMS) values during the neural recording experiments. See **Fig 8**for a firing rate analysis of the same data. Both spontaneous firing rate and barb deflection firing rate measured with an electrode in the coxa were higher than with an electrode in the femur.

The spontaneous firing rate measured with the coxa electrode was 31.9 ± 18.8 Hz (s.d.), significantly higher than that measured with the femur electrode, at 4.0 ± 3.3 Hz (coxa intact, p = 0.004, df = 14, N = 16) and 4.4 ± 2.8 Hz (coxa detached, p = 0.001, df = 14, N = 16). There was not a significant difference in the spontaneous rates measured with the femur electrode between the coxa attached versus unattached conditions (p = 0.77, df = 14, N = 16).

When the tibial barbs were manually deflected, firing rates always unequivocally increased, regardless of electrode configuration. Though the robust increase in firing rate from spontaneous resting state during tibial barb deflection was obvious in all cases (and indeed why the cockroach leg preparation makes such a compelling educational preparation), the coxa electrode measured a significantly higher firing rate, at 84.8 ± 30.7 Hz, compared to the femur electrode conditions (39.9 ± 8.8 Hz with the coxa intact, p = 0.001, df = 14, N = 16 and 37.7 ± 7.1 Hz with the coxa detached, p = 0.008, df = 14, N = 16). The higher firing rate upon barb deflection measured from the coxa electrode is likely due to the higher intrinsic spontaneous rate as well as possible activation of joint and stretch sensory receptors (hair plates and chordotonal organs) in the coxa due to the pressure of touching the tibial barbs [14, 19]. There was not a significant difference in firing rate upon barb deflection between the coxa intact and coxa detached conditions measured with the femur electrode (p = 0.6, df = 14, N = 16).

Given that four nerves (nerve numbers 3, 4, 5, and 6) innervate the coxa, but only one of these nerves subsequently innervates the femur and tibia (nerve number 5) [16–18], with one electrode in the coxa and the other electrode in the tibial/tarsus joint, it is possible the electrical activity of four nerves is being recorded simultaneously. However, with one electrode in the femur and the other in the tibia/tarsus joint, only one nerve is being recorded, nerve 5. This offers a potential explanation for why the spontaneous firing rate is lower with an electrode in the femur, due to the smaller neural population.

Hence, an autotomy-removed leg vs. a coxa-removed leg has minimal effect on the usefulness of the leg preparation for teaching neurophysiology rate coding: brushing the barbs causes an increase in spiking activity, and this increase in spiking activity is proportional to the strength of the tactile stimulus. The other important observation, that an electrode in the coxa has a higher spontaneous firing rate compared to an electrode in the femur, may be of interest to a class studying the design of neural interfaces, principles of selecting the proper ground location, recording from different populations, etc.

**Fig 9:**
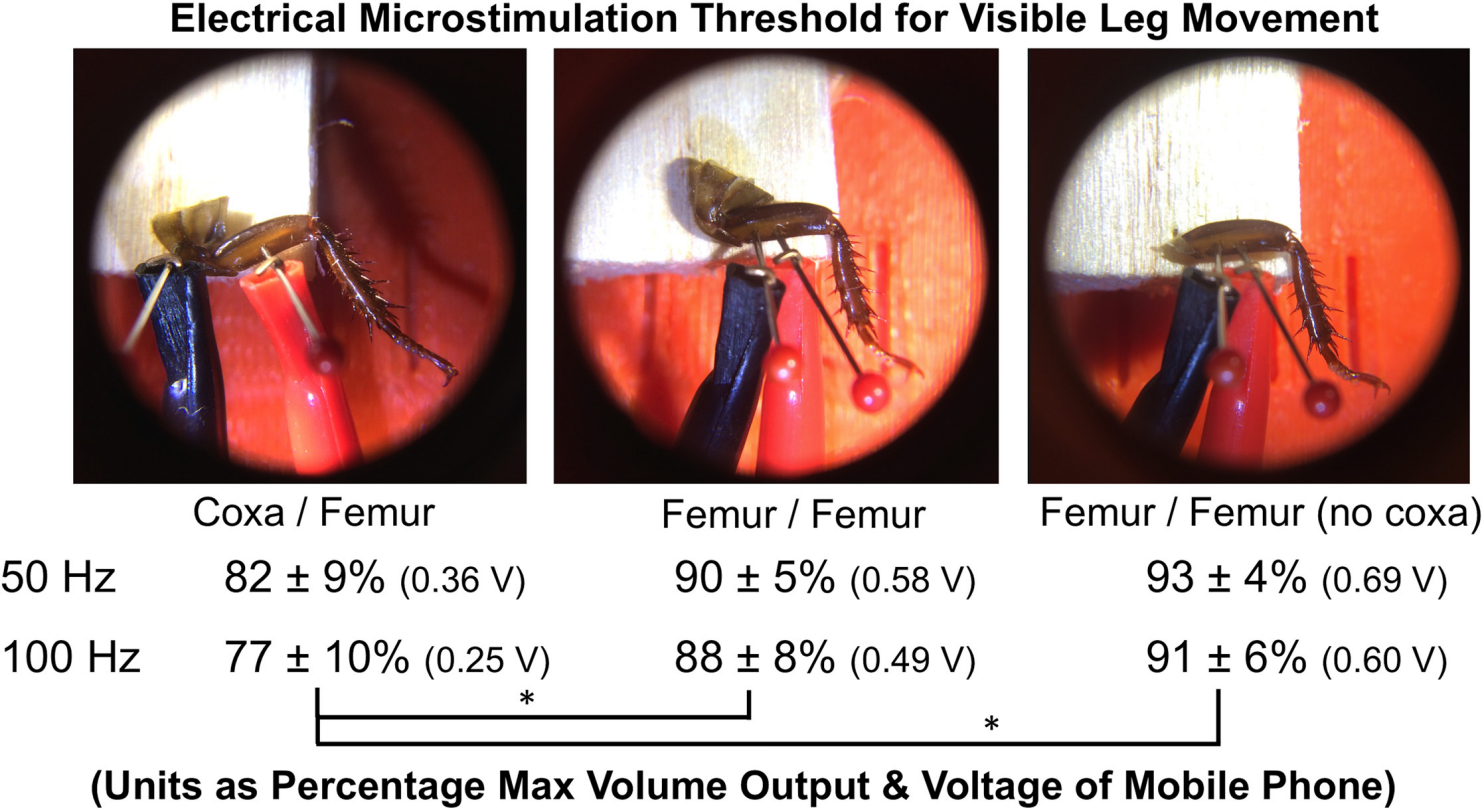
Microstimulation Thresholds for Eliciting Cockroach Leg Deflection. Electrodes were first placed in the coxa and femur and connected to modified audio cables plugged into an iPhone 5s running the “Tone Generator” App. The volume necessary (threshold) to cause visible leg movement at two electrical sine wave frequencies was then measured. The coxa electrode was subsequently moved alongside the femur electrode, and thresholds tested again. Finally, the coxa was removed with forceps, and thresholds measured similarly. Thresholds were significantly higher with both electrodes in the femur (bipolar configuration) versus one electrode in the coxa and the other in the femur (monopolar configuration). There was not a significant difference in thresholds between the coxa present and coxa absent conditions with both electrodes in the femur. Thresholds were measured in 8 legs during the three conditions and then averaged. Voltage output of iPhone was measured with an oscilloscope and is also reported next to threshold values. * = p<0.05.

See **Fig 9**for a summary of the microstimulation experiments. When one electrode is in the coxa and the other electrode is in the femur, this can be considered analogous to “monopolar” stimulation, where the stimulating electrode is distant from the return ground electrode. With both electrodes in the femur, this can be considered “bipolar” stimulation, where both the stimulating and return electrodes are in nearby tissue. It is known in the microstimulation literature that bipolar stimulation has higher stimulation thresholds due to more localized current spread between the two electrode pairs [25]. The experiments performed here verify such results. The “bipolar” configuration, with both electrodes in the femur, had a 10% higher activation threshold of movement compared to the monopolar configuration (p = 0.021 (coxa intact), p = 0.0055 (coxa absent), df = 14, N = 16). The presence or absence of the coxa did not affect the threshold in the bipolar (both electrodes in the femur) stimulation experiments (p = 0.17, df = 14, N = 16).

Important to note is that, with the leg removed via autotomy, while thresholds are higher, movements can still be elicited. In the context of a classroom demonstration, these higher thresholds are manageable and do not detract from the teaching experience.

### Number of Molts and Intermolt Time

**Fig 10:**
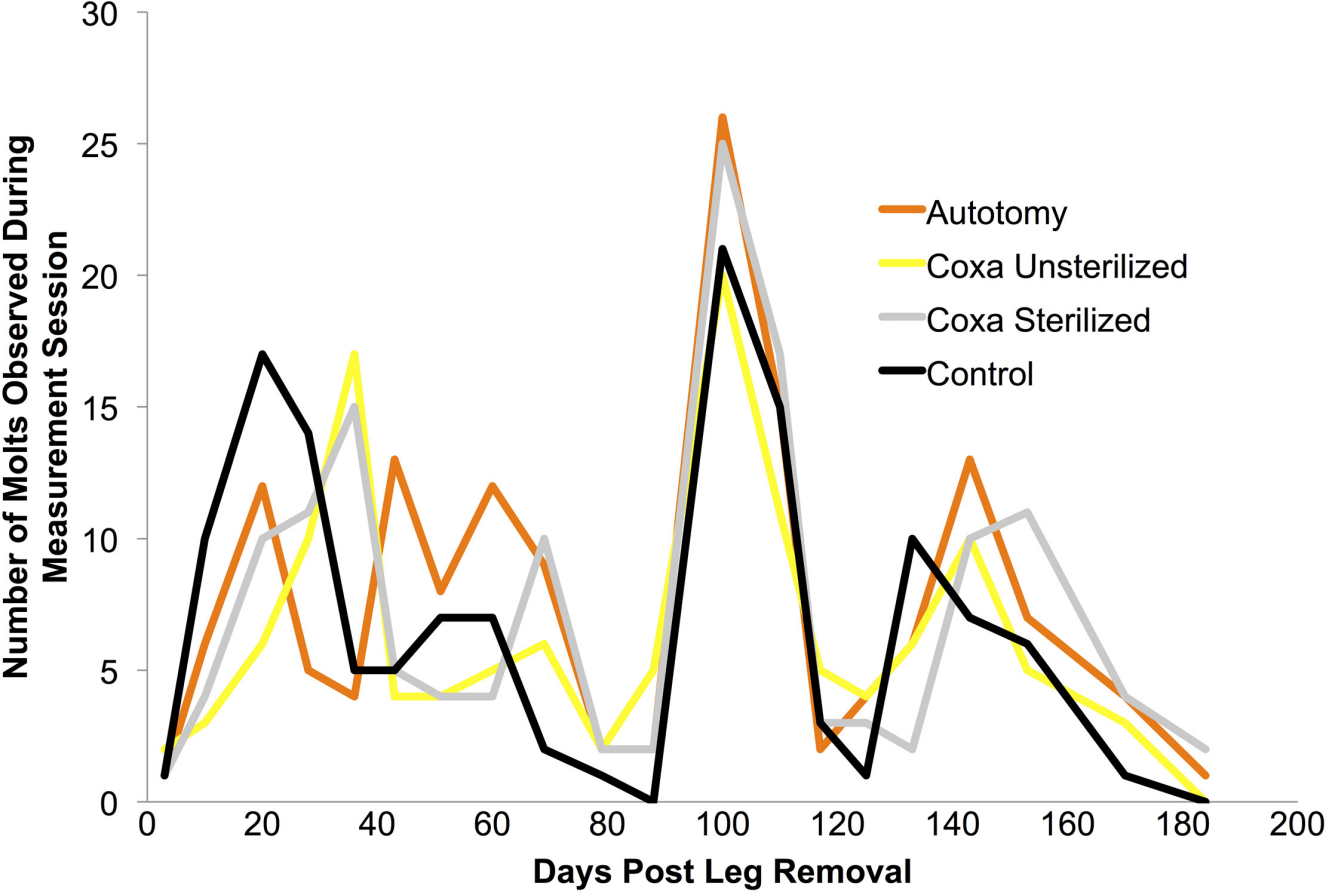
Number of molts observed during each measurement period in the four groups. Differences between groups was not apparent, though the “group synchronization” of peak molting at day 100 and 150 may deserve further examination in future work.

Total autotomy, coxa sterilized, and coxa unsterilized molts were 152, 128, and 145, respectively, not varying notably from the number of control molts (140) observed. No apparent differences were seen in the time course of molts between groups on given observation points (see **Fig 10**). Interestingly, there appeared to be synchronization in peak molting at day 100 and day 150 in all groups. Such synchronization may merit further investigation in future studies unrelated to limb loss.

Even though coxa-cut cockroaches were molting at a frequency and time course comparable to the autotomy and control groups, the regenerated leg did not begin appearing until much later following limb amputation. This is hypothesized to be due to the healing process of the coxa, which is likely delaying the regeneration of the leg in the coxa-cut group (See **Fig 4**for an example of the “coxa healing molt”).

Though the growth of the new leg probably puts an increased metabolic load on the cockroach, longer intermolt times were not detected in the experimental animals versus the controls with unremoved legs. However, the time difference in molting time between leg-removed cockroaches and controls previously reported in the literature is quite small (only a 1 day delay) and was studied in newly hatched cockroaches that molt approximately every week [10], shorter than the 30–60 days between molts in the juvenile cockroaches in this study.

Of note is to clarify that this work does not determine the average number of molts needed in the coxa and autotomy groups before the regenerated leg first appears. Such a measurement requires maintaining the identity of each individual cockroach throughout the duration of the experiment, which encountered difficulties during pilot studies. For example, during an observation session, if five cockroaches in a sub-group terrarium had molted, matching molted cockroaches to their “exoskeleton” sheaths was largely guesswork. Housing and caring for 216 cockroaches in individual cages was not feasible given lab resources, and, moreover, may be detrimental to individual cockroaches’ health [26].

However, given the previous literature as well as the experiments done here, a testable hypothesis is that a coxa-cut leg, due to the slower healing process, needs 2–4 molts for a regenerated new leg to appear, whereas, as the literature already reveals, the autotomy-removed leg needs 1–2 molts for a regenerated leg to appear.

### Comparison with Previous Literature

In previous cockroach leg regeneration studies, limbs were always cut at the autotomization points [7, 9–10, 27–29]. When the legs are removed at the trochanter/femur autotomy joint in young cockroaches, the literature notes three effects that occur dependent on the remaining intermolt time. If the animal is very near molting, a “coagulated hemolymph” healing wound appears on the subsequent molt that looks similar to the original wound. If the animal is past a certain critical period but has enough time to heal the wound, a papilla with a smooth coxa and trochanter appears without a regenerated leg. If the animal has its leg removed before a “critical period”, a regenerated leg appears on the subsequent molt. The data presented here in this manuscript are in agreement with the previous literature on autotomy, with the first two responses observed before 36 days in the autotomy group, and the third response occurring in almost all autotomy cockroaches after 36–43 days. In the coxa-cut-group, however, many molts (71%, or 193 of 273 observed molts) revealed only a healing coxa (1^st^ and 2^nd^ response) throughout the duration of the study, indicating a much slower, more variable, healing process.

The only manuscript found that addresses severing of legs at mid-coxa level was by Joseph Kunkel [8]. Though the majority of his study was on removal of the leg at the femur/trochanter autotomy joint, he did in passing note in 4^th^ instar *Periplaneta americana* that:

*“Amputation between the coxa and trochanter does not result in any regeneration at the subsequent molt*. *Some molting delay occurs which may be related to the substantial injury and blood loss caused by amputation at this non-autotomy point*.*”*

Studied systematically here, more substantial healing indeed needs to occur in the coxa-cut group before a new leg can appear, and the new leg is ~2.5x smaller than a regenerated leg that results from autotomy leg removal. It would be interesting to study and understand whether a coxa-cut cockroach 1) first regrows the coxa and then subsequently grows a leg inside the regenerated coxa, or 2) simultaneously regrows the coxa and new leg. Careful microscopic studies would reveal which of these two hypotheses is likely to bear out.

For example, the wound healing and leg regrowth process has been well characterized in second stage *americana* instars injected with tritiated thymidine to monitor cell division after a trochanter/femur autotomy joint leg removal [13, 30]. Initially, the leaking hemolymph dries and forms a temporary seal, which then permanently seals as cells near the wound enlarge and migrate underneath the dried hemolymph. After this, the epidermis near the wound separates and forms a blastema, and this blastema then forms a new leg. Since the coxa-cuts in this manuscript are a more dramatic injury than autotomy removal, such a healing process may utilize different regeneration mechanisms (perhaps due to loss of epidermal stem cells in the coxa) that explain the longer time for a new leg to appear in the coxa-cut group.

## Conclusion

Given our research group’s work using cockroach leg neurophysiology as an educational preparation that is often replicated by teachers in classrooms, we hereby recommend that leg removal proceed via autotomy at the trochanter/femur joint in lieu of cutting at the coxa, as autotomy-removed legs regenerate more rapidly and reliably than coxa-cut legs, and the same neurophysiology experiments can still be undertaken on autotomy-removed legs.

The autotomy-removed cockroach leg is thus a near ideal biological preparation for its utility in classrooms for 1) teaching introductory principles of neurophysiology, and 2) regeneration studies. After doing the neural recording experiments, students interested in development can measure and observe the leg regrowth, which can serve to teach about contemporary biological research in tissue regeneration and its relevance to human afflictions. Such experiments are increasingly easier to carry out and improve upon as open source science tools become more and more available [31].

## References

[1] Marzullo TC, Gage GJ. 2012 The spikerbox: a low cost, open-source bioamplifier for increasing public participation in neuroscience inquiry. Plos ONE, 7, e30837 10.1371/journal.pone.0030837 22470415 PMC3310049

[2] Linder TM, Palka J. 1992 A student apparatus for recording action potentials in cockroach legs. Am J Physiol, 262, s18–22. 1616063 10.1152/advances.1992.262.6.S18

[3] Ramos RL, Moiseff A, Brumberg JC. 2007 Utility and versatility of extracellular recordings from the cockroach for neurophysiological instruction and demonstration. J Undergrad Neurosci Educ 5:A28–A34. 23494074 PMC3592644

[4] Maginnis TL. 2006 The costs of autotomy and regeneration in animals: a review and framework for future research. Behavioral Ecology, 17, 857–872.

[5] Réaumur RA. 1734–1742. Mémoires pour servir à l'histoire des insects, Paris, de l'imprimerie royale.

[6] Brindley HH. 1897 On the regeneration of the legs in the blatidae. Prec. Gen. Meet. Sci. Bus. Zool. Soc. London, 903–916.

[7] Bodenstein D. 1955 Contributions to the problem of regeneration in insects. Journal of Experimental Zoology, 129, 209–224.

[8] Kunkel JG. 1977 Cockroach molting. II The nature of regeneration-induced delay of molting hormone secretion. Biol Bull, 153, 145–62. 889943 10.2307/1540698

[9] Woodruff L, Seamans L. 1939 The rate of regeneration in the german roach. Annals of the Entomological Society of America, 32, 589–600.

[10] O'Farrell AR, Stock A. 1953 Regeneration and the moulting cycle in blattella germanica l. I. Single regeneration initiated during the first instar. Aust j biol sci, 6, 485–500. 13093536 10.1071/bi9530485

[11] Willis ER, Riser GR, Louis M. 1958 Observations on reproduction and development in cockroaches. Annals of the Entomological Society of America, 51, 53–69.

[12] Kunkel JG. 1966 Development and the availability of food in the german cockroach, blattela germanica. J. Insect. Physiol., 12, 227–235.

[13] Truby RR. 1983 Blastema formation and cell division during cockroach limb regeneration. J Embryol Exp Morphol, 75, 151–64. 6886608

[14] Cruse H, Dürr V, Schilling M, Schmitz J. 2009 Principles of insect locomotion In: Spatial temporal patterns for action-oriented perception in roving robots. Arena P, Patanè L (Eds); Cognitive Systems Monographs, 1, Berlin: Springer: 43–96.

[15] Pringle JWS. 1939 The motor mechanism of the insect leg. J. Exp. Biol. 16: 220–231;

[16] Nijenhuis ED, Dresden D. 1955 On the topographical anatomy of the nervous system of the mesothoracic leg of the american cockroach (periplaneta americana). I. Proc. K. Ned. Akad. Wet., C58, 121–130.

[17] Nijenhuis ED, Dresden D. 1955 On the topographical anatomy of the nervous system of the mesothoracic leg of the american cockroach (periplaneta americana). II. Proc. K. Ned. Akad. Wet., C58, 131–136.

[18] Zill SN, Underwood MA, Rowley JC III, Moran DT. 1980 A somatotopic organization of groups of afferents in the insect peripheral nerves. Brain Research. 198: 253–269. 7407598 10.1016/0006-8993(80)90743-x

[19] Chapman KM. 1965 Campaniform sensilla on the tactile spines of the legs of the cockroach. J. Exp. Biol. 42: 191–203. 14323763 10.1242/jeb.42.2.191

[20] Spence AJ, Revzen S, Seipel J, Mullens C, Full RJ. 2010 Insects running on elastic surfaces. J Exp Biol, 213, 1907–20. 10.1242/jeb.042515 20472778

[21] Harley CM, English BA, Ritzmann RE. 2009 Characterization of obstacle negotiation behaviors in the cockroach, blaberus discoidalis. J Exp Biol, 212, 1463–76. 10.1242/jeb.028381 19411540

[22] Watson JT, Ritzmann RE, Pollack AJ. 2002 Control of climbing behavior in the cockroach, blaberus discoidalis. II. Motor Activities associated with joint movement. J Comp Physiol a Neuroethol Sens Neural Behav Physiol. 188(1):55–69. 11935230 10.1007/s00359-002-0278-x

[23] Dagda RK, Thalhauser RM, Dagda R, Marzullo TC, Gage GJ. 2013 Using crickets to introduce neurophysiology to early undergraduate students. J Undergrad Neurosci Educ 12: A66–A74. 24319394 PMC3852874

[24] Mongeau JM, McRae B, Jusufi A, Birkmeyer P, Hoover AM, Fearing R, et al 2012 Rapid inversion: running animals and robots swing like a pendulum under ledges. PLoS ONE. 7(6):e38003 10.1371/journal.pone.0038003 22701594 PMC3368944

[25] Merrill DR, Bikson M, Jefferys JG. 2005 Electrical stimulation of excitable tissue: design of efficacious and safe protocols. J Neurosci Methods 141, pp. 171–98. 15661300 10.1016/j.jneumeth.2004.10.020

[26] Bell WJ, Roth LM, Nalepa CA. 2007 Cockroaches: ecology, behavior, and natural history Johns Hopkins University Press.

[27] Marks EP, Leopold RA. 1970 Cockroach leg regeneration: effects of ecdysterone in vitro. Science, 167, 61–62. 5409478 10.1126/science.167.3914.61

[28] Cowden RR, Bodenstein D. 1961 A cytochemical investigation of striated muscle differentiation in regenerating limbs of the roach, periplanata americana. Embryologia, 6, 36–50.

[29] Penzlin H. 1963 Uber die regeneration bei schaben (blattaria) i. Das regenerationsvermogen und die genese des regenerats. Roux' arehivffir enwieklungsmechanik, 154, 434–465. 10.1007/BF00576568 28354197

[30] Truby PR. 1985 Separation of wound healing from regeneration in the cockroach leg. J embryol exp morphol, 85, 177–90. 3989448

[31] Baden T, Chagas AM, Gage GJ, Marzullo TC, Prieto-Godino LL, Euler T. 2015 Open Labware: 3-D printing your own lab equipment. PLoS Biol. 3 20;13(3). 10.1371/journal.pbio.1002086 PMC4368627 25794301

